# Supplementary material for: IFNγ signaling in cytotoxic T cells restricts anti-tumor responses by inhibiting the maintenance and diversity of intra-tumoral stem-like T cells
Source: Nat Commun. 2023 Jan 19;14:321. doi: 10.1038/s41467-023-35948-9 (PMC9852295; doi:10.1038/s41467-023-35948-9)
Supplement: Supplementary file 1 — Supplementary Information [file 41467_2023_35948_MOESM1_ESM.pdf]

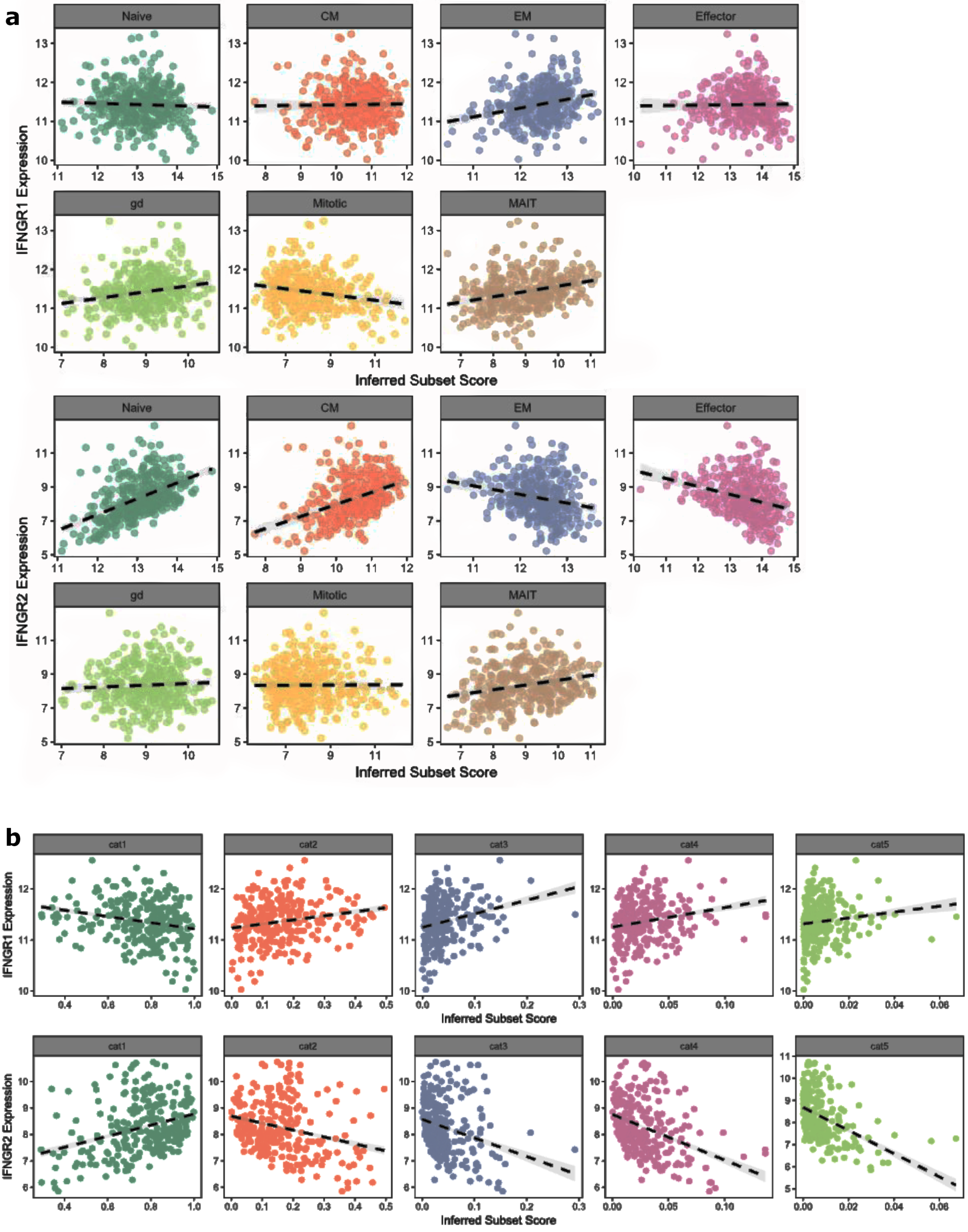
a
Naive
CM
EM
Effector
gd
Mitotic
MAIT
IFNGR1 Expression
IFNGR2 Expression
Inferred Subset Score
b
cat1
cat2
cat3
cat4
cat5
IFNGR1 Expression
IFNGR2 Expression
Inferred Subset Score

Figure S1. IFNγR expression correlations with inferred subsets in bulk RNAseq. **a**- Relationship between IFNγR expression and T cell subset scoring. T cell Subset score has been calculated on bulk RNAseq samples to estimate proportions of each of the CD8 T cell subsets in that sample. Graphs show the level of IFNγR1 (upper panels) and IFNγR2 (lower panels) transcripts over the different subsets scoring.
b- Relationship between IFNγR expression and clonal size scoring. Cat1-5 represents the different clonal sizes, with Cat1 being small clones <0.1% of the repertoire whilst Cat5 is the hyperexpanded clones >2.5%. Graphs show the level of IFNγR1 (upper panels) and IFNγR2 (lower panels) transcripts over the different subsets scoring.

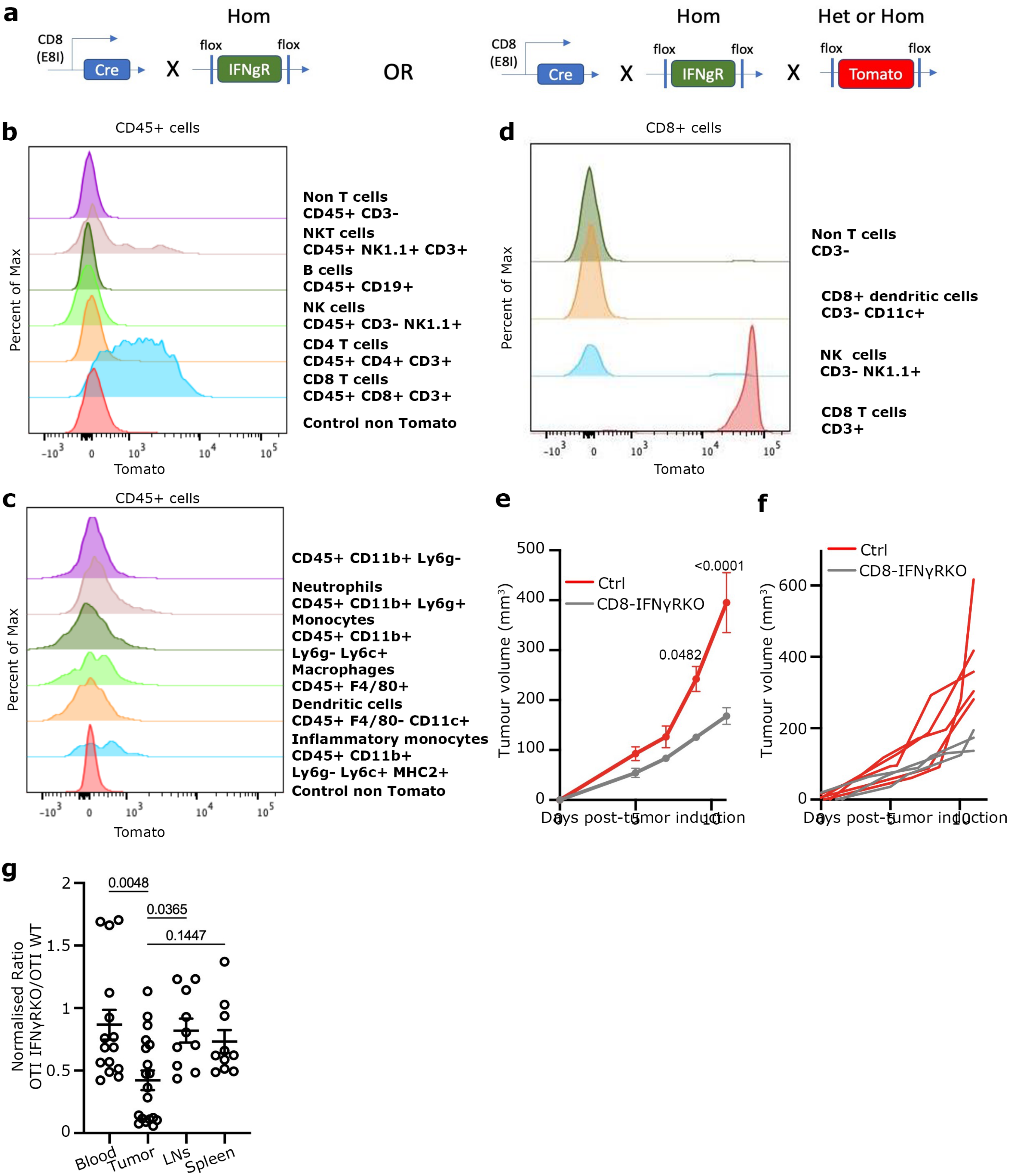

Figure S2: Inhibition of IFNγ sensing by CD8 T cells; method and relevance for MC38 tumour growth and T cell homing to tumor.
(**a-d**) CD8-IFNγRKO mouse model to ablate IFNγR1 specifically in CD8 T cells. **a**- Diagram of the mouse model. CD8-Cre mice were crossed with IFNγR1 flox mice. In some experiments, mice were also crossed with ROSA-Tomato (Homozygous or Heterozygous). Controls used in experiments were either CD8-Cre only or IFNγR1 flox with or without ROSA-Tomato. (**b-d**) Characterization of CD8-IFNγRKO mouse using Tomato expression as a readout of Cre expression specificity. Splenocytes from B16-OVA tumor-bearing mice were analyzed by flow cytometry. (**b-c**) Splenocytes were labelled with the indicated antibodies to delineate the main immune subsets within the CD45⁺ lineage and the histogram shows Tomato expression within those subsets. Top panel shows lymphoid lineages, bottom panel shows myeloid lineages. **d**- Splenocytes were labelled with the indicated antibodies to delineate the main CD8 expressing immune subsets and the histogram shows Tomato expression within those subsets, demonstrating the specificity of the mouse model for mature CD8 T cells. (**e-f**) Control (red) and CD8- IFNγRKO (grey) mice were engrafted with MC38 tumors. **e**- The graph shows the average tumor volume over time. Data shows Mean with SEM (n=5). Mixed-effects model analysis with Šidák's multiple comparison test **f**- The graph shows the tumor volume of individual mice over time. Every line is a mouse (n=5). **g**- In vitro activated WT or IFNγRKO OTI were ad-mixed and injected in B16-OVA tumor-bearing mice. After 24h, the ratio between IFNγRKO and WT OTI was assessed in tumors, LNs, and spleens by flow cytometry. Every dot is a mouse (n= 15 for blood, n= 19 for tumor, n= 10 for LN and n= 10 for spleen) from 2 independent experiments. One-way Anova with Tukey's multiple comparisons test.

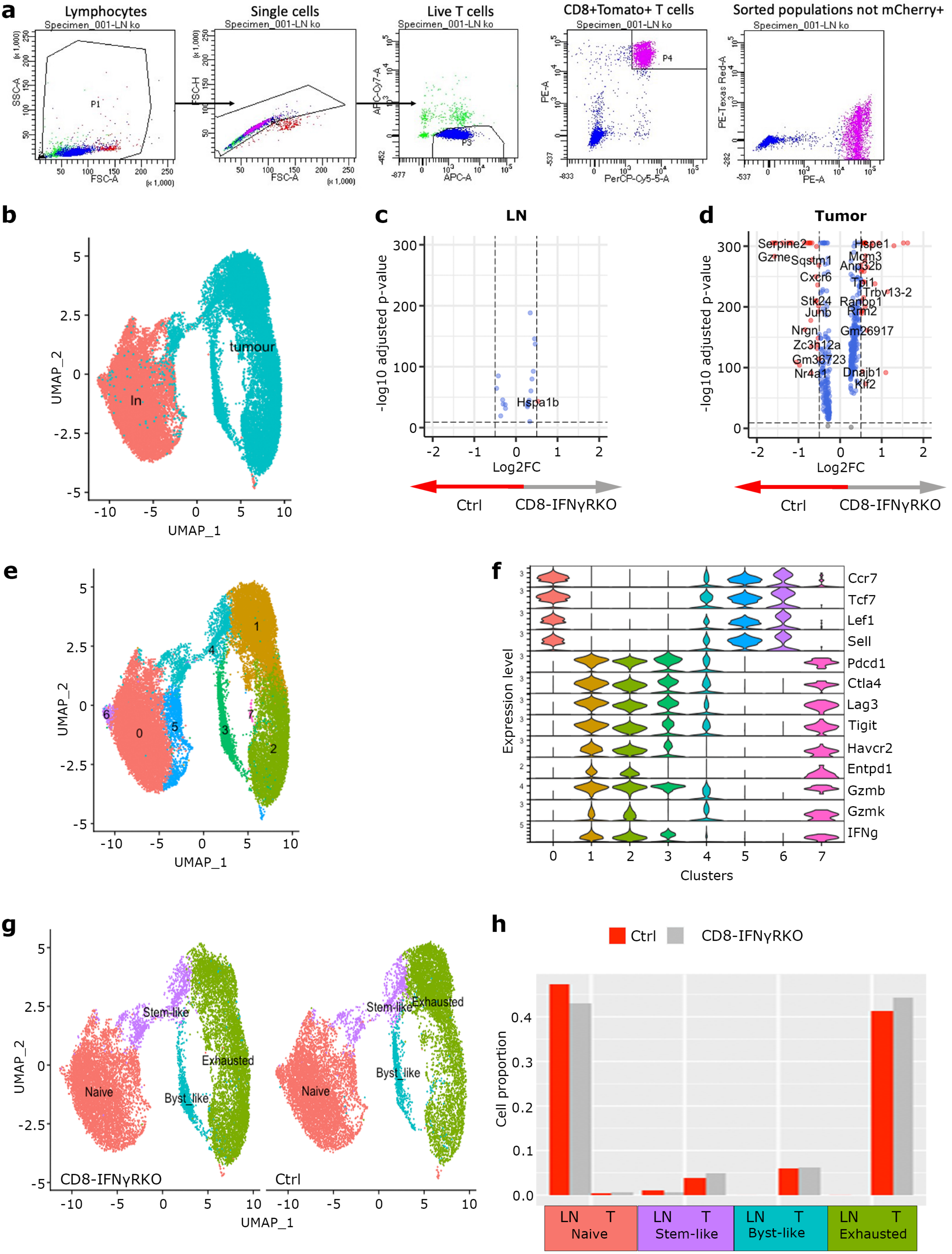
a
Lymphocytes
Single cells
Live T cells
CD8+Tomato+ T cells
Sorted populations not mCherry+
b
tumour
ln
UMAP_1
UMAP_2
c
LN
Hspa1b
-log10 adjusted p-value
Log2FC
Ctrl
CD8-IFNγRKO
d
Tumor
Serpine2
Gzme
Sqstm1
Cxcr6
Stk24
Junb
Nrgn
Zc3h12a
Gm36723
Nr4a1
Hspe1
Mcm3
Anp32b
Tpi1
Trbv13-2
Ranbp1
Rrm2
Gm26917
Dnajb1
Klf2
e
f
Ccr7
Tcf7
Lef1
Sell
Pdcd1
Ctla4
Lag3
Tigit
Havcr2
Entpd1
Gzmb
Gzmk
IFNg
Expression level
Clusters
g
Stem-like
Exhausted
Naive
Byst_like
CD8-IFNγRKO
Ctrl
h
Ctrl
CD8-IFNγRKO
Cell proportion
LN T Naive
LN T Stem-like
LN T Byst-like
LN T Exhausted

Figure S3: Unbiased characterization of CD8 T cells in lymph nodes and tumors of Control and CD8-IFNγRKO mice. Control (n=3) and CD8-IFNγRKO (n=3) mice were engrafted with B16-OVA tumors. $CD45^{+}CD3^{+}CD8^{+}Tomato^{+}$ cells were sorted from tumors and draining lymph nodes and subjected to scRNA-seq analysis. **a**- Gating strategy for CD8 T cell sort. **b**- UMAP embeddings of merged scRNA-seq profiles from tumor (tumour) and lymph node (ln) CD8 T cells. (**c-d**) Volcano plot representing Differentially Expressed Genes between control and CD8-IFNγRKO CD8 T cells from LN (**c**) and tumor (**d**). Blue dots: genes with an adjusted p value<0.05; Red dots: genes with log2 (fold-change) value >0.5 or <-05 and an adjusted p value<0.05. Wilcoxon rank sum test. **e**- Graph-based clustering of identified 8 clusters. **f**- Violin plots show the expression level (y-axes) of selected exhaustion markers (*Pdcd1* (encoding PD-1), *Entpd1* (encoding CD39), *Ctla4*, *Lag3, Tigit*, and *Havcr2* (encoding Tim-3)), effector markers (*IFNγ, Gzmk, Gzmb*), and naïve-like markers (*Ccr7, Tcf7, Lef1*, and *Sell* (encoding for CD62L), in each of the identified clusters (x-axes). **g**- Graph-based clustering of the assembled cell states according to the gene combination identified in (**f**). **h**- The bar plots show the percentages of CD8 T cells from Control (red) and CD8-IFNγRKO (grey) mice that were found in each cell state from LN and tumors.

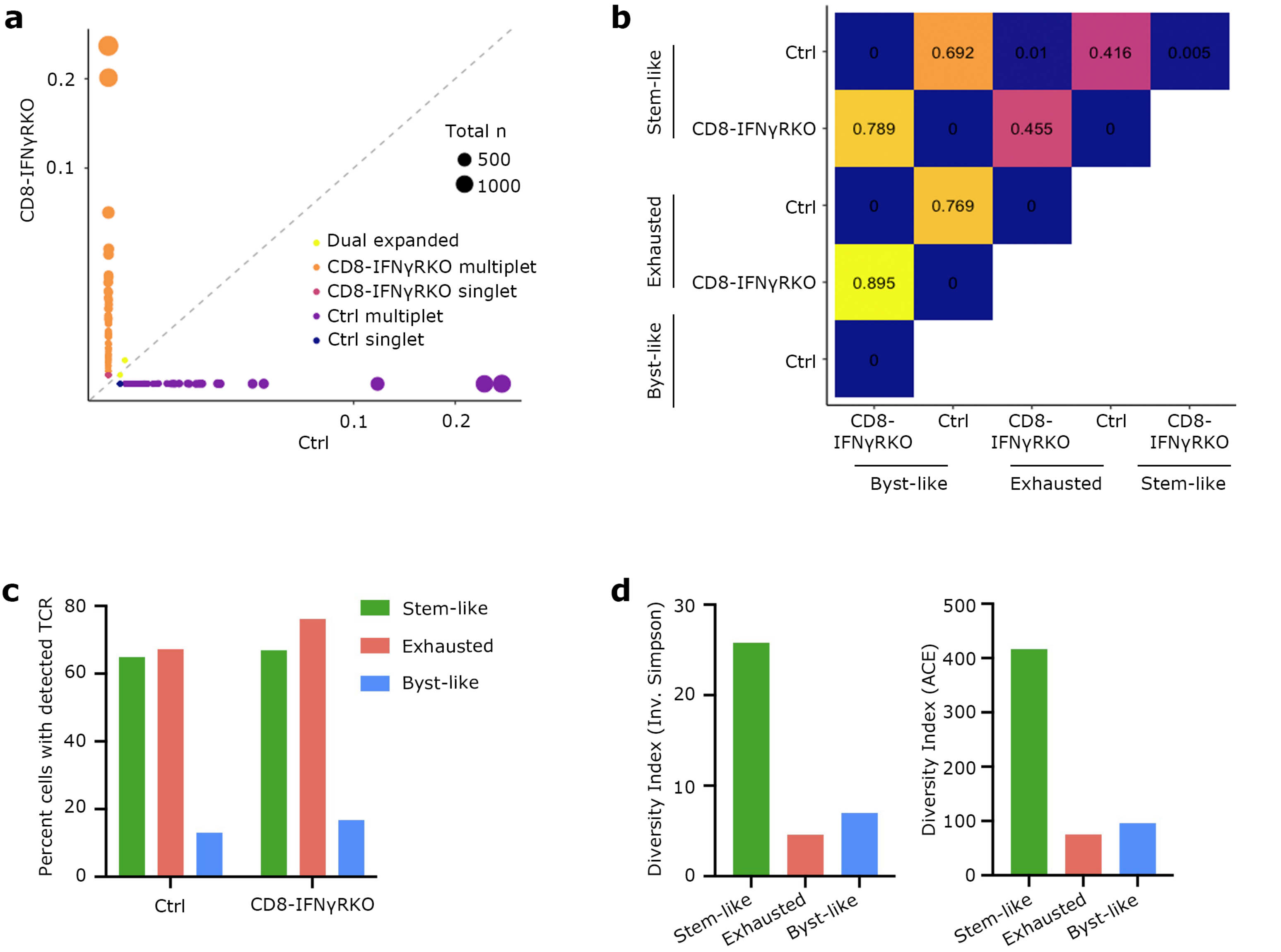

Figure S4: Features of Single Cell TCR sequencing of intra-tumoral TILs in B16-OVA melanoma. Control (n=3) and CD8-IFNγRKO (n=3) mice were engrafted with B16-OVA tumors. $CD45^{+}CD3^{+}CD8^{+}Tomato^{+}$ cells were sorted from tumors and subjected to scTCR- and scRNA-seq a nalysis. **a**- Scatter plot shows the comparison of clonotypes between Control and CD8-IFNγRKO TILs. The axes represent the relative proportion of the clonotype across all clonotypes. The dot size represents the total number of clones between the x- and y-axis. **b**- The graph represents the clonal similarity between the different cell states for Control and CD8-IFNγRKO TILs. Similarity values correspond to the overlap coefficient, defined as the overlap of clonotypes scaled to the length of unique clonotypes in the smaller sample. **c**- Bar plot shows the percentage of cells for which a complete TCR (alpha and beta chain) was detected for Control and CD8-IFNγRKO TILs over the different cell states. **d**- Bar plot shows the diversity index (left graph = Inv. Simpson; right graph = ACE) of the different cell states, regardless of the genotype.

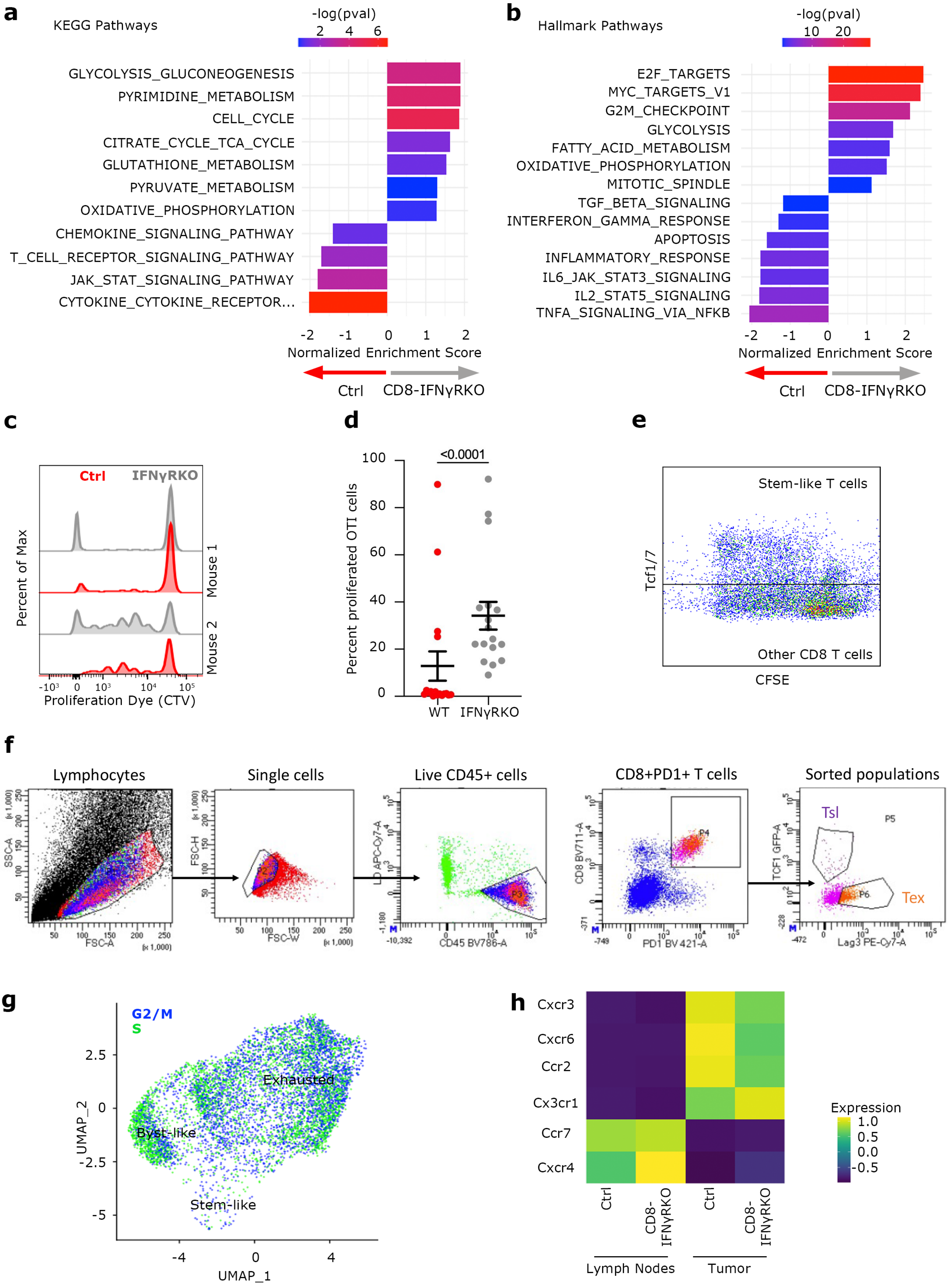
a
KEGG Pathways
-log(pval)
GLYCOLYSIS_GLUCONEOGENESIS
PYRIMIDINE_METABOLISM
CELL_CYCLE
CITRATE_CYCLE_TCA_CYCLE
GLUTATHIONE_METABOLISM
PYRUVATE_METABOLISM
OXIDATIVE_PHOSPHORYLATION
CHEMOKINE_SIGNALING_PATHWAY
T_CELL_RECEPTOR_SIGNALING_PATHWAY
JAK_STAT_SIGNALING_PATHWAY
CYTOKINE_CYTOKINE_RECEPTOR...
Normalized Enrichment Score
Ctrl
CD8-IFNγRKO
b
Hallmark Pathways
-log(pval)
E2F_TARGETS
MYC_TARGETS_V1
G2M_CHECKPOINT
GLYCOLYSIS
FATTY_ACID_METABOLISM
OXIDATIVE_PHOSPHORYLATION
MITOTIC_SPINDLE
TGF_BETA_SIGNALING
INTERFERON_GAMMA_RESPONSE
APOPTOSIS
INFLAMMATORY_RESPONSE
IL6_JAK_STAT3_SIGNALING
IL2_STAT5_SIGNALING
TNFA_SIGNALING_VIA_NFKB
Normalized Enrichment Score
Ctrl
CD8-IFNγRKO
c
Ctrl
IFNγRKO
Percent of Max
Mouse 1
Mouse 2
Proliferation Dye (CTV)
d
<0.0001
Percent proliferated OTI cells
WT
IFNγRKO
e
Stem-like T cells
Other CD8 T cells
Tcf1/7
CFSE
f
Lymphocytes
Single cells
Live CD45+ cells
CD8+PD1+ T cells
Sorted populations
Tsl
Tex
g
G2/M
S
Exhausted
Byst-like
Stem-like
UMAP_1
UMAP_2
h
Cxcr3
Cxcr6
Ccr2
Cx3cr1
Ccr7
Cxcr4
Expression
Ctrl
CD8-IFNγRKO
Ctrl
CD8-IFNγRKO
Lymph Nodes
Tumor

Figure S5: Further characteristics of Ctrl and CD8-IFNγRKO T cells. (**a-b,g**) Control (n=3) and CD8-IFNγRKO (n=3) mice were engrafted with B16-OVA tumors. CD45+CD3+CD8+Tomato+ cells were sorted from tumors and subjected to scRNA-seq analysis. (**a-b**) Bar plot shows the NES of chosen KEGG (**a**) and Hallmark (**b**) pathways up- and down-regulated in CD8-IFNγRKO vs control Bystander-like TILs. Colors correspond to adjusted p-values, derived from the fgsea R package (**c-d**) Control (red) and IFNγRKO (grey) OTI T cells were labelled with the proliferation dye CTV, ad-mixed (1:1), and transferred in WT mice bearing B16-OVA. Three to five days after OTI transfer, CTV dilution was assessed by flow cytometry. **c**- Representative histograms showing CTV dilution of 2 mice. **d**- Graph shows the percent of cells that displayed CTV dilution in tumors. Data are from 3 independent experiments (n = 17). Data shows Mean with SEM. Unpaired t test. **e**- Ex vivo restimulation in the presence of blocking IFNγ antibody of isolated TILs from tumor-bearing WT mice. Cells were labelled with CFSE at day 0, stained for CD8, Tcf1/7, and PD-1 at the indicated time points, and analyzed by flow cytometry. Representative dot plot showing CFSE dilution against Tcf1/7 expression 4 days after ex vivo culture. **f**- Gating strategy for intratumoral stem-like and exhausted CD8 T cell sort using live cells> CD45$^+$(BV785)>CD8$^+$(BV711)>PD-1$^+$(BV421) and Tcf1$^+$(GFP), Lag-3$^-$(PE-Cy7) for stem-like T cells or tcf1$^-$Lag-3$^+$ for exhausted T cells. **g**- UMAP visualization of the distribution of cell cycle scoring (G2/M phase: blue; S phase: green). **h**- Control and CD8-IFNγRKO mice were engrafted with B16-OVA tumors. CD45+CD3+CD8+Tomato+ cells were sorted from tumor-draining lymph nodes or tumors, and subjected to scRNA-seq analysis. Heatmap shows the relative average expression (z-score) of selected chemokine receptors for control and CD8-IFNγRKO CD8 T cells from tumor-draining lymph nodes and tumors.

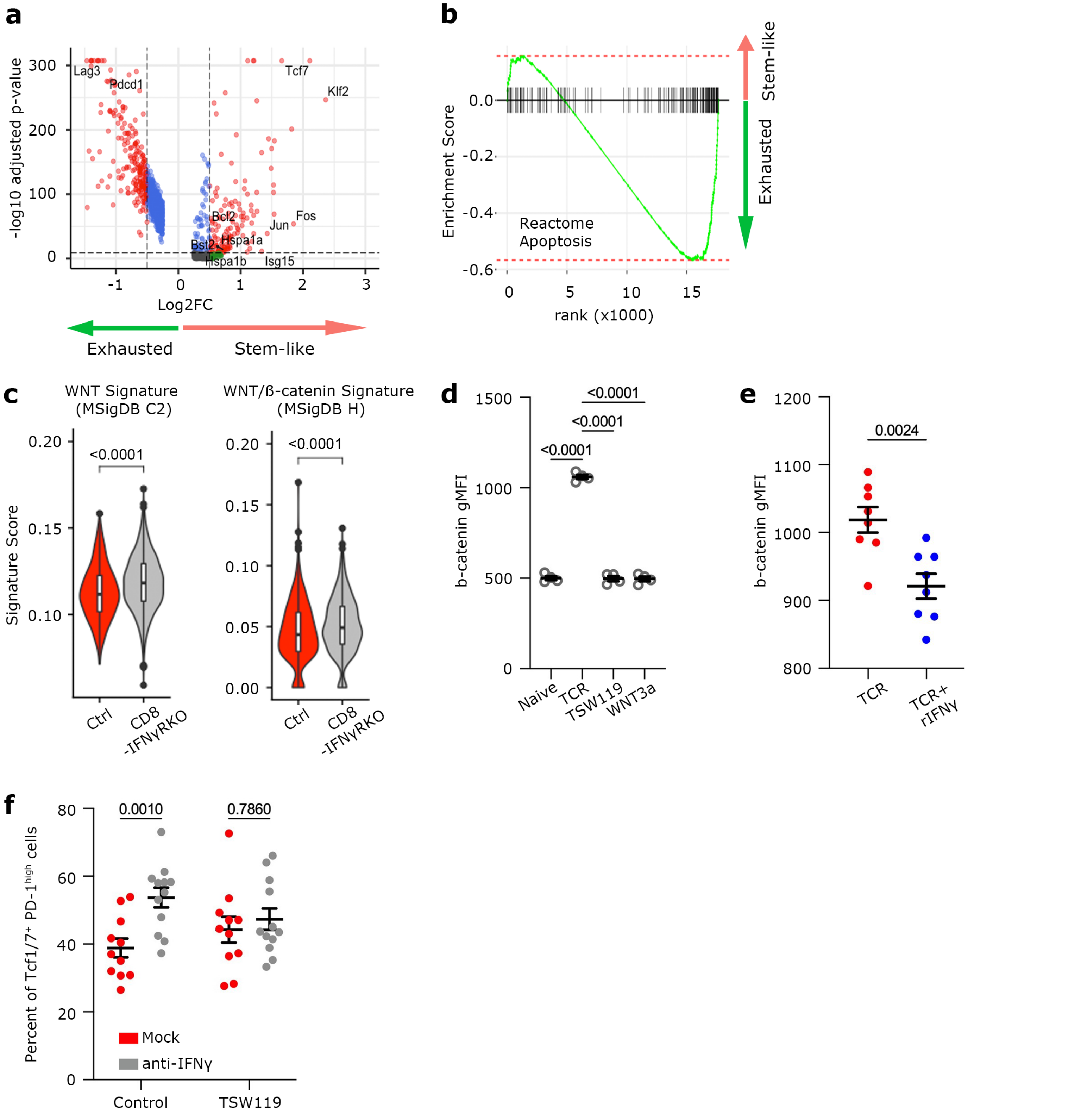

Figure S6: Relationship between IFNγ sensing, stemness and β-catenin. Control (n=3) and CD8-IFNγRKO (n=3) mice were engrafted with B16-OVA tumors. CD45+CD3+CD8+Tomato+ cells were sorted from tumors and subjected to scRNA-seq analysis. **a**- Volcano plot representing Differentially Expressed Genes between Exhausted and Stem-like CD8 T cells. Green dots: genes with log2 (fold-change) value >0.5 or <-05; Blue dots: genes with an adjusted p value<0.05; Red dots: genes with log2 (fold-change) value >0.5 or <-05 and an adjusted p value<0.05. Wilcoxon rank sum test. **b**- Enrichment Score of the Reactome apoptosis pathway between Exhausted and Stem-like CD8 T cells. **c**- Violin Plots show the quantification of WNT and β-catenin signatures of Control (red) and CD8-IFNγRKO (grey) cells within the stem-like state. Box plots indicate median (middle line), 25th, 75th percentile (box). Two-sided Wilcoxon signed-rank test. (n = 15706 total). **d**- Naïve CD8 T cells from Lymph nodes were treated with anti-CD3 and anti-CD28 (TCR), the glycogen synthase kinase 3 beta (GSK3β) inhibitor TWS119 or the WNT agonist WNT3a for 6 hours. β-catenin expression was assessed by flow cytometry.Every dot is a sample (n=4) from 2 independent experiments. Data shows Mean with SEM. Unpaired t test. **e-** Naïve CD8 T cellsfrom Lymph nodes were pretreated with IFNγ when indicated for 16 hours before being stimulated with anti-CD3 and anti-CD28 (TCR) for 6 hours. β-catenin expression was assessed by flow cytometry. Every dot is a sample (n=8) from 2 independent experiments. Data shows Mean with SEM. Unpaired t test. **f**- Naïve CD8 T cells from Lymph nodes were stimulated with anti-CD3 and anti-CD28 in the presence of IL-2. When indicated, cells were treated with anti-IFNγ and the GSK3β inhibitor TWS119. After 4 to 5 days, cells were harvested and stained for CD45, CD8, Tcf1/7, and PD-1. The graph shows the percentage of PD1high Tcf1/7+ stem-like CD8 T cells following anti-CD3 and anti-CD28 stimulation in the presence (grey) or absence (red) of anti-IFNγ. Every dot is a sample (n=11 control; n=12 anti-IFNγ) from 3 independent experiments). Data shows Mean with SEM. Unpaired t test.

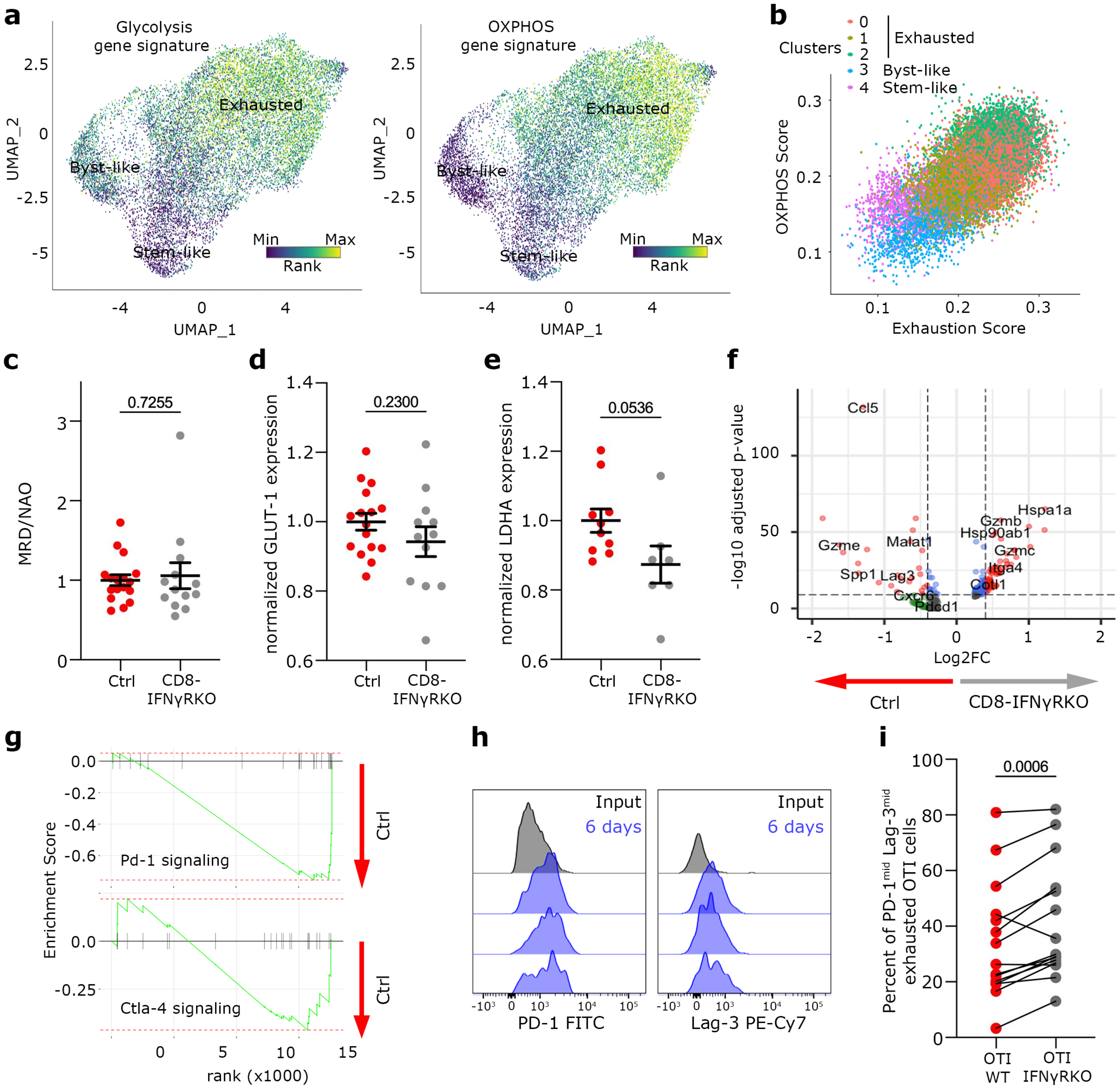

Figure S7: IFNγ signaling in intra-tumoral CD8 T cells reinforces exhaustion.(**a-b, f-g**) Control (n=3) and CD8-IFNγRKO (n=3) mice were engrafted with B16-OVA tumors. CD45$^+$CD3$^+$CD8$^+$Tomato$^+$ cells were sorted from tumors and subjected to scRNA-seq analysis. **a**- UMAP visualization of Glycolysis gene signature (left) and OXPHOS gene signature (right). **b**- The dot plot shows OXPHOS score according to Exhaustion Score. Colors correspond to original clusters. Pearson Correlation = 0.64. (**c-e**) Control (red) and CD8-IFNγRKO (grey) mice were engrafted with B16-OVA, and metabolic status of exhausted (PD-1$^+$ LAG3$^+$) CD8 T cells was analyzed by flow cytometry after 12 days. **c**- Graph shows mitochondrial function (MDR, MitoTracker deep red) per mitochondrial mass (NAO, Nonyl Acridine Orange). Each dot is a mouse (Control n=17 and CD8-IFNγRKO n=13) from 3 independent experiments. Graph shows mean +SEM. Unpaired *t* test. **d**- Graph shows normalized protein level expression of GLUT1.Each dot is a mouse (Control n=16 and CD8-IFNγRKO n=12) from 3 independent experiments. Graph shows mean +SEM. Unpaired *t* test. **e**- Graph representing the anaerobic glycolysis level using normalized LDHA expression as a readout. Each dot is a mouse (Control n=10 and CD8-IFNγRKO n=7) from 2 independent experiments. Graph shows mean +SEM. Unpaired *t* test. **f**- Volcano plot representing Differentially Expressed Genes between control and CD8-IFNγRKO CD8 T cells in the Memory-Bystander-like state. Green dots: genes with log2 (fold-change) value >0.5 or <-05; Blue dots: genes with an adjusted p value<0.05; Red dots: genes with log2 (fold-change) value >0.5 or <-05 and an adjusted p value<0.05. Total variables = 341. **g**- Enrichment Score of PD-1 (upper panel) and CTLA-4 (lower panel) between CD8-IFNγRKO and control TILs in the Memory-Bystander-like state. (**h-i**) In vitro-generated stem-like WT (red) and IFNγRKO (grey) OTI cells were injected at a 1:1 ratio in tumor-bearing mice. After 6 days, tumors were harvested, and OTI cells were analyzed by flow cytometry. Data is from 2 independent experiments. **h**- Representative histogram showing increased PD-1 and Lag-3 expression 6 days after transfer. Each blue line represent a mouse. **i**- Graphs show the percentage of exhausted CD8 T cells with intermediate expression of PD-1 and Lag-3. Lines link control and IFNγRKO OTI from the same mouse. Each dot is a mouse (n=12) from 3 experiments. Data shows Mean +/- SEM. Paired t test.
