# Supplementary material for: IFNγ signaling in cytotoxic T cells restricts anti-tumor responses by inhibiting the maintenance and diversity of intra-tumoral stem-like T cells
Source: Nat Commun. 2023 Jan 19;14:321. doi: 10.1038/s41467-023-35948-9 (PMC9852295; doi:10.1038/s41467-023-35948-9)
Supplement: Supplementary file 3 — Reporting Summary [file 41467_2023_35948_MOESM3_ESM.pdf]

nature portfolio

Corresponding author(s): Audrey Gerard

Last updated by author(s): 2022/10/16

Reporting Summary

Nature Portfolio wishes to improve the reproducibility of the work that we publish. This form provides structure for consistency and transparency in reporting. For further information on Nature Portfolio policies, see our Editorial Policies and the Editorial Policy Checklist.

Statistics

For all statistical analyses, confirm that the following items are present in the figure legend, table legend, main text, or Methods section.

n/a	Confirmed	
☐	☒	The exact sample size (*n*) for each experimental group/condition, given as a discrete number and unit of measurement
☐	☒	A statement on whether measurements were taken from distinct samples or whether the same sample was measured repeatedly
☐	☒	The statistical test(s) used AND whether they are one- or two-sided<br>*Only common tests should be described solely by name; describe more complex techniques in the Methods section.*
☒	☐	A description of all covariates tested
☒	☐	A description of any assumptions or corrections, such as tests of normality and adjustment for multiple comparisons
☐	☒	A full description of the statistical parameters including central tendency (e.g. means) or other basic estimates (e.g. regression coefficient) AND variation (e.g. standard deviation) or associated estimates of uncertainty (e.g. confidence intervals)
☐	☒	For null hypothesis testing, the test statistic (e.g. *F*, *t*, *r*) with confidence intervals, effect sizes, degrees of freedom and *P* value noted<br>*Give P values as exact values whenever suitable.*
☒	☐	For Bayesian analysis, information on the choice of priors and Markov chain Monte Carlo settings
☒	☐	For hierarchical and complex designs, identification of the appropriate level for tests and full reporting of outcomes
☐	☒	Estimates of effect sizes (e.g. Cohen's *d*, Pearson's *r*), indicating how they were calculated

Our web collection on statistics for biologists contains articles on many of the points above.

Software and code

Policy information about availability of computer code

Data collection	Flow cytometry data was recorded using BDFACSDiva (v8.0) software<br>No new bioinformatics tools or algorithms were created
Data analysis	Flow cytometry data was analyzed using Flowjo (v10.4.2).<br>Single cell RNA sequencing data was analyzed using R (v 4.1.3), Seurat (v4.0.6), UCell (v.1.3), Monocle3 (v.1.0.0), Vision (v.3.0.0), fgsea, scRepertoire (v.1.3.5), DESeq2(v.1.38.1), scProportionTest.<br>Data was visualized using Graphpad (V8.4.1, Prism software), EnhancedVolcano (v1.12.0), GGplot2(v3.3.5) and ggpubr(v0.5.0).<br>Imaging was analyzed with Imaris (Bitplane – version 9.6)

For manuscripts utilizing custom algorithms or software that are central to the research but not yet described in published literature, software must be made available to editors and reviewers. We strongly encourage code deposition in a community repository (e.g. GitHub). See the Nature Portfolio guidelines for submitting code & software for further information.

Data

Policy information about availability of data

All manuscripts must include a data availability statement. This statement should provide the following information, where applicable:

- Accession codes, unique identifiers, or web links for publicly available datasets
- A description of any restrictions on data availability
- For clinical datasets or third party data, please ensure that the statement adheres to our policy

The mouse scRNAseq and scTCRseq data generated in this study have been deposited in the GEO database under accession code GSE221118 (https://www.ncbi.nlm.nih.gov/geo/query/acc.cgi?acc=GSE221118). Datasets reused in this study: EGAS00001005507 (https://ega-archive.org/studies/EGAS00001005507), EGAS00001004081 (https://ega-archive.org/search-results.php?query=EGAS00001004081), GSE120575 (https://www.ncbi.nlm.nih.gov/geo/query/acc.cgi?acc=GSE120575), GSE99254 (https://www.ncbi.nlm.nih.gov/geo/query/acc.cgi?acc=GSE99254). All data are included in the Supplemental Information or available from the authors upon reasonable requests, as are unique reagents used in this Article. The raw numbers for charts and graphs are available in the Source Data file whenever possible.

Human research participants

Policy information about studies involving human research participants and Sex and Gender in Research.

Reporting on sex and gender	n/a
Population characteristics	n/a
Recruitment	n/a
Ethics oversight	n/a

Note that full information on the approval of the study protocol must also be provided in the manuscript.

Field-specific reporting

Please select the one below that is the best fit for your research. If you are not sure, read the appropriate sections before making your selection.

☒ Life sciences ☐ Behavioural & social sciences ☐ Ecological, evolutionary & environmental sciences

For a reference copy of the document with all sections, see nature.com/documents/nr-reporting-summary-flat.pdf

Life sciences study design

All studies must disclose on these points even when the disclosure is negative.

Sample size	Sample size was estimated based on prior experience and complexity. Whenever possible, we used g*power to calculate sample size. Sample size was computed to detect difference of 50% in a treated group with a standard deviation of 20%, false positive of 0.05, and power of 0.80. Sample size was therefore calculated to be between 7-8. Where smaller changes were expected, sample sizes were increased accordingly.
Data exclusions	no data exclusion
Replication	All findings were replicated in two to four separate experiments (as specified in legends), with the exception of the single cell RNA sequencing experiment, in which data was aggregated from multiple separate biological samples as indicated in the figure legends and methods section. All separate experiments yielded comparable trends and results.
Randomization	For mouse experiments involving multiple genotypes, we used littermates, and controlled that gender and age was similar between all groups. Whenever possible, comparison was done between populations within the same mouse, for which randomization is not required. For re-analysis of human data, we used the assignments of clusters from the original study. In Figure 1, human samples were already allocated to the groups (non-progressing, progressing, pre-treatment, post-treatment).
Blinding	No blinding was performed during mouse experiments when all mice within an experiment received identical treatments. No subjective scoring methods which would require blinding were used. Most experiments were performed and repeated independently by two researchers. Flow cytometry data were collected in an automatic and unbiased manner.

Reporting for specific materials, systems and methods

We require information from authors about some types of materials, experimental systems and methods used in many studies. Here, indicate whether each material, system or method listed is relevant to your study. If you are not sure if a list item applies to your research, read the appropriate section before selecting a response.

Materials & experimental systems

n/a	Involved in the study
☐	☒ Antibodies
☐	☒ Eukaryotic cell lines
☒	☐ Palaeontology and archaeology
☐	☒ Animals and other organisms
☒	☐ Clinical data
☒	☐ Dual use research of concern

Methods

n/a	Involved in the study
☒	☐ ChIP-seq
☐	☒ Flow cytometry
☒	☐ MRI-based neuroimaging

Antibodies

Antibodies used

Antibodies Conjugate Dilution Supplier Cat number
Anti-CD3 145-2C11 N/A Armenian Hamster IgG 1/250 (2ug/mL final) Biolegend 100302
Anti-CD28 37.51 N/A Syrian Hamster IgG 1/250 (2ug/mL final) Biolegend 102102
anti-IFNg XMG1.2 N/A Rat IgG1, k 1/700 (10ug/mL final) BioXcell BE0055
CD8a EPR21769 A647 rabbit 1/200 Abcam ab237365
anti-rabbit polyclonal A647 donkey 1/400 Biolegend 406414
CD45 30-F11 BV785 Rat IgG2b, κ 1/200 Biolegend 103149
CD45 30-F11 BV650 Rat IgG2b, κ 1/200 Biolegend 103151
CD8a 53-6.7 PerCP.Cy5.5 Rat IgG2a, κ 1/200 Biolegend 100734
CD8a 53-6.7 BV711 Rat IgG2a, κ 1/200 Biolegend 100747
CD8a 53-6.7 BV605 Rat IgG2a, κ 1/200 Biolegend 100744
CD44 IM7 A700 Rat IgG2b, κ 1/200 Biolegend 103025
CD44 IM7 A647 Rat IgG2b, κ 1/200 Biolegend 103017
CD44 IM7 BV785 Rat IgG2b, κ 1/200 Biolegend 103041
CD69 H1.2F3 Armenian Hamster IgG PE 1/200 Biolegend 104507
F4/80 BM8 APC Rat IgG2a, κ 1/200 Biolegend 123115
CD3 17A2 PE/Cy7 Rat IgG2b, κ 1/200 Biolegend 100220
CD3 17A2 PerCP.Cy5.5 Rat IgG2b, κ 1/200 Biolegend 100218
NK1.1 PK136 BV605 Mouse IgG2a, κ 1/200 Biolegend 108739
CD19 6D5 A488 Rat IgG2a, κ 1/200 Biolegend 115524
CD19 6D5 PerCP.Cy5.5 Rat IgG2a, κ 1/200 Biolegend 152406
CD4 RM4-5 BV421 Rat IgG2a, κ 1/200 Biolegend 100543
CD11b M1/70 BV421 Rat IgG2b, κ 1/200 Biolegend 101235
CD11c N418 BV605 Armenian Hamster IgG 1/200 Biolegend 117333
Ly6C HK1.4 PE/Cy7 Rat IgG2c, κ 1/200 Biolegend 128018
Ly6G 1A8 FITC Rat IgG2a, κ 1/200 Biolegend 127606
MHC-II M5/114.15.2 A700 Rat IgG2b, κ 1/200 Biolegend 107622
CD45.1 A20 BV421 Mouse (A.SW) IgG2a, κ 1/200 Biolegend 110731
CD45.2 104 FITC Mouse (SJL) IgG2a, κ 1/200 Biolegend 109805
LAMP1 1D4B A700 Rat IgG2a, κ 1/200 Biolegend 121627
LAG3 C9B7W PE/Cy7 Rat IgG1, κ 1/200 Biolegend 125225
LAG3 C9B7W BV785 Rat IgG1, κ 1/200 Biolegend 125219
PD1 29F.1A12 BV421 Rat IgG2a, κ 1/200 Biolegend 135217
PD1 29F.1A12 APC Rat IgG2a, κ 1/200 Biolegend 135209
PD1 29F.1A12 FITC Rat IgG2a, κ 1/200 Biolegend 135213
PD1 29F.1A12 BV605 Rat IgG2a, κ 1/200 Biolegend 135219
TIM3 RMT3-23 APC Rat IgG2a, κ 1/200 Biolegend 119705
TIGIT 1G9 BV421 Mouse IgG1, κ 1/200 Biolegend 142111
IFNg XMG1.2 BV421 Rat IgG1, κ 1/100 Biolegend 505829
TNFa MP6-XT22 APC Rat IgG1, κ 1/100 Biolegend 506307
GLUT1 EPR3915 A405 rabbit 1/100 Abcam ab210438
GLUT1 polyclonal A647 rabbit 1/100 Novus Biol NB110-39113
ATPb 3D5 A488 Mouse IgG1 1/100 Abcam 14730
GADPH 14C10 A647 rabbit IgG 1/100 Cell Signalling 3907S
LDHA E9 NIR mouse 1/50. SCBT sc-137243
Tcf1/7 C63D9 A488 rabbit 1/100 Cell Signalling 6444S
Tcf1/7 C63D9 A647 rabbit 1/100 Cell Signalling 6709S
pan-beta-catenin 15B8 e660 Mouse / IgG1, kappa 1/100 Invitrogen 50-2567-42
cleaved caspase 3 C92-605 A647 rabbit 1/100 BD Bioscience 560626

Validation

All antibodies were obtained from commercial vendors and we based specificity on descriptions and information provided in

corresponding Data Sheets available and provided by the Manufacturers.
Dilution optimization was performed on splenocytes or immune cells isolated from tumours.
Biolegend (human and mouse)- Flow Cytometry Reagents:-Specificity testing of 1-3 target cell types with either single- or multi-color analysis (including positive and negative cell types).-Once specificity is confirmed, each new lot must perform with similar intensity to the in-date reference lot. Brightness (MFI) is evaluated from both positive and negative populations.-Each lot product is validated by QC testing with a series of titration dilutions. https://www.biolegend.com/en-us/quality/quality-control

BioXCell: Advanced Binding Validation utilizes a library of recombinant proteins and bioassay expertise to validate that each lot of applicable InVivoPlusTM antibody binds strongly and specifically to its target antigen.
AbCam: When validating antibodies for flow cytometry, we are careful to optimize every step of the staining protocol, including fixation, permeabilization, and washing. To do this, our scientists review the available literature to understand which cell types and conditions are best suited to validate specific antibodies. We include relevant controls, routinely running unstained, positive, negative, isotype, viability, Fc-blocking, fluorescence minus one (FMO), and single-staining controls. For an FMO control, we stain all our samples with fluorescent conjugates except the one that is being tested. This shows the contribution of the other fluorescent conjugates in the signal of the unlabeled channel. This control is important for determining non-specific binding of an antibody.

Novus Biol: We recognize the need to enhance antibody validation for our customers. In addition to our current validation procedures, Novus will now implement several new methods for antibody validation, in accordance with recommendations instigated by the International Working Group for Antibody Validation (IWGAV). Genetic Strategy Validation- Expression of the target protein is compared before and after knockout or knockdown using CRISPR/CAS9 or siRNA/shRNA. If protein expression following knockout or knockdown is substantially reduced, then antibody specificity is ensured.
Orthogonal Validation- The target protein is examined with an antibody independent strategy and compared with results from an antibody-dependent strategy. A correlation between these two strategies indicates specificity between the antibody and its target protein.
Independent Antibody Validation- The data generated using several antibodies (ideally targeting different epitopes) in the same protein is compared (e.g. molecular weight and cellular localization). Consistent results imply antibody selectivity to the target protein.
Expression of Tagged Proteins Validation- A tagged protein is used as a standard for comparison in Western blotting and/or immunocytochemistry (ICC). For example, if the distribution of the tagged protein overlaps with the immunofluorescence signal, then antibody specificity is confirmed.
Biological Strategies Validation- These strategies use defined biological or chemical modulation of protein expression to demonstrate antibody specificity to the target protein. The data is compared across multiple cell lines including positive and negative expressing cells, and multiple species, if applicable.

Cell Signalling: To ensure our antibodies will work in your experiment, we adhere to the Hallmarks of Antibody Validation™, six complementary strategies that can be used to determine the functionality, specificity, and sensitivity of an antibody in any given assay. CST adapted the work by Uhlen, et. al., ("A Proposal for Validation of Antibodies." Nature Methods (2016)) to build the Hallmarks of Antibody Validation, based on our decades of experience as an antibody manufacturer and our dedication to reproducible science.

Invitrogen: Part 1—Target specificity verification
This helps ensure the antibody will bind to the correct target. Our antibodies are being tested using at least one of the following methods to ensure proper functionality in researcher's experiments:
Knockout—expression testing using CRISPR-Cas9 cell models
Knockdown—expression testing using RNAi to knockdown gene of interest
Independent antibody verification (IAV)—measurement of target expression is performed using two differentially raised antibodies recognizing the same protein target
Cell treatment—detecting downstream events following cell treatment
Relative expression—using naturally occurring variable expression to confirm specificity
Neutralization—functional blocking of protein activity by antibody binding
Peptide array—using arrays to test reactivity against known protein modifications
SNAP-ChIP™—using SNAP-ChIP to test reactivity against known protein modifications
Immunoprecipitation-Mass Spectrometry (IP-MS)—testing using immunoprecipitation followed by mass spectrometry to identify antibody targets
Part 2—Functional application validation
These tests help ensure the antibody works in a particular application(s) of interest, which may include
(but are not limited to):
Western blotting
Flow cytometry
ChIP
Immunofluorescence imaging
Immunohistochemistry

BD: Antibody specificity BD Biosciences identifies key targets of interest in scientific research and develops its own specific antibodies or collaborates with top research scientists around the world to license their antibodies. We then transform these antibodies into flow cytometry reagents by conjugating them to a broad portfolio of high-performing dyes, including our vastly popular portfolio of BD Horizon Brilliant™ Dyes. A world-class team of research scientists helps ensure that these reagents work reliably and consistently for flow cytometry applications. The specificity is confirmed using multiple methodologies that may include a combination of flow cytometry, immunofluorescence, immunohistochemistry or western blot to test staining on a combination of primary cells, cell lines or transfectant models. All flow cytometry reagents are titrated on the relevant positive or negative cells. To save time and cell samples for researchers, test size reagents are bottled at an optimal concentration with the best signal-to-noise ratio on relevant models during the product development. To ensure consistent performance from lot-to-lot, each reagent is bottled to match the

previous lot MFI. You can look up the Certificate of Analysis and the concentration of test-size human reagents from specific lots via the Concentration Lookup page or BD Regulatory Documents. Technical data sheets provide data generated on the relevant primary model at this optimal concentration based on a titration curve. QC data on any lot of reagent can be requested through ResearchApplications@bd.com. Quality control Our dedication to rigorous testing and high-quality control standards means that you can use our reagents in your research with the utmost confidence. All BD reagent facilities, including our California Design Center at San Diego, our manufacturing facilities in Tatabanya (Hungary) and San Diego (USA), and our California instrument facility (Manufacturing and Design Centers) at San Jose, are approved and registered according to the internationally defined ISO 9001 standard. Once our research and development (R&D) team completes evaluation of a new product, the developed process is transferred to our manufacturing teams, including Quality Control. Our manufacturing process adheres to standard operating procedures (SOPs) and guidelines, which are based on ISO requirements and are strictly followed, helping ensure that reagents provide consistent results to help give you assurance of experimental success and confidence in your research. Quality control testing of newly manufactured lots is performed side-by-side with a previously accepted lot as a control, helping to assure that performance of the new lot is both reliable and consistent. https://www.bdbiosciences.com/en-us/products/reagents/flow-cytometry-reagents/research-reagents/quality-and-reproducibility

Eukaryotic cell lines

Policy information about cell lines and Sex and Gender in Research

Cell line source(s)	B16 Tyr-/- expresssing mCherry and Ovalbumin (B16OVA) was provided by Ed Roberts, Beatson Institute, University of Glasgow<br>The murine colon carcinoma MC-38 was provided by Matthew Krummel
Authentication	Both Cell lines used were not authenticated. OVA expression of B16 melanoma cells has been confirmed using in vitro activation assays with transgenic T cells (OT-I respectively). mCherry expression in B16 melanoma cells has been confirmed by microscopy. Tyrosine KO (Tyr-/-) inhibits melanin formation and could be confirmed macroscopically by looking at the colour of the B16.
Mycoplasma contamination	Both cell lines have been regularly tested for Mycoplasma contamination and tested negative
Commonly misidentified lines<br>(See ICLAC register)	No commonly misidentified cell lines were used in this study.

Animals and other research organisms

Policy information about studies involving animals; ARRIVE guidelines recommended for reporting animal research, and Sex and Gender in Research

Laboratory animals	species: mus musculus,<br>strains: C57bl/6, CD45.1, OT-I, CD8a-Cre, IFNgRflox (CD119flox), ROSA-Tomato, IFNgRKO, UCB-GFP , Tcf7f/f-GFP<br>sex: male and female<br>age: 6-14 weeks
Wild animals	wild animals were not used in this study
Reporting on sex	Both genders were used in experiments. Males and Females were equally distributed throughout conditions. We did not initially find any difference between males and females and therefore did not take gender into account for analysis.
Field-collected samples	study did not involve field-collected samples
Ethics oversight	All experiments involving mice were performed in agreement with the United Kingdom Scientific Procedures Act of 1986

Note that full information on the approval of the study protocol must also be provided in the manuscript.

Flow Cytometry

Plots

Confirm that:

- [x] The axis labels state the marker and fluorochrome used (e.g. CD4-FITC).
- [x] The axis scales are clearly visible. Include numbers along axes only for bottom left plot of group (a 'group' is an analysis of identical markers).
- [x] All plots are contour plots with outliers or pseudocolor plots.
- [x] A numerical value for number of cells or percentage (with statistics) is provided.

Methodology

Sample preparation	See method section of manuscript. In some experiments, T cells were isolated from the lymph nodes of 6 to 12 weeks-old mice. In other experiments, tumors and draining lymph nodes (LNs) were harvested, measured, and weighed. Tumors were

	then dilacerated, incubated 30 minutes at 37C in 5% CO_2 in R10 containing Liberase TL at 1mg/mL (Roche) and DNase I at 10ug/mL (Roche), and run through a 70µm cell strainer (Falcon). Cells were either undergoing Ficoll to obtain live immune cells or directly used after for further experiments. LNs were directly smashed through a 70µm cell strainer and resuspended in PBS.
Instrument	FACSAria™ II (BD) was used for sorting and Fortessa X-20 for analysis
Software	Data collection: BDFACSDiva (v8.0) software<br>Data analysis: Flowjo v10.8.1
Cell population abundance	Populations were sorted at >95% purity, determined by flow cytometric analysis of post-sort samples
Gating strategy	Cells were sorted based on expression of CD45, CD3, CD8, and Tomato for scRNAseq. Cells were sorted based on expression of CD45, CD3, CD8, Tcf7, PD-1, Lag3 for ex-vivo restimulation.

[x] Tick this box to confirm that a figure exemplifying the gating strategy is provided in the Supplementary Information.
