# Supplementary material for: IFNγ signaling in cytotoxic T cells restricts anti-tumor responses by inhibiting the maintenance and diversity of intra-tumoral stem-like T cells
Source: Nat Commun. 2023 Jan 19;14:321. doi: 10.1038/s41467-023-35948-9 (PMC9852295; doi:10.1038/s41467-023-35948-9)
Supplement: Supplementary file 4 — Description of Additional Supplementary Files [file 41467_2023_35948_MOESM4_ESM.docx]

**Description of Additional Supplementary Files**

Supplementary Dataset 1: List of genes that correlated and anti-correlate with IFNγR1 expression in CD8 T cells from metastatic melanoma patients after checkpoint blockade. Normalized IFNGR1 expression was used as a continuous variable in DESeq2. Wald test. S

upplementary Dataset 2: List of genes that correlated and anti-correlate with IFNγR2 expression in CD8 T cells from metastatic melanoma patients after checkpoint blockade. Normalized IFNGR2 expression was used as a continuous variable in DESeq2. Wald test.

Supplementary Dataset 3: Differentially expressed genes between CD8- IFNγRKO and control CD8 T cells within the different cell states. Significant differentially expressed genes between clusters were identified using the “FindAllMarkers” function, Wilcoxon test and selecting markers expressed in at least 25% of cells.

Supplementary Dataset 4: Antibodies used in this study

Supplementary Dataset 5: Gene signatures used in this study.
